# Aberrant right posterior hepatic artery originating from the gastroduodenal artery with a concomitant replaced left hepatic artery: a rare clinical variant

**DOI:** 10.1007/s00276-025-03814-6

**Published:** 2026-01-29

**Authors:** Murat Emre Reis, Yavuz Selim Angın, Orxhan Ulfanov, Murat Ulaş, Mehmet Kılıç, Elif Gündoğdu

**Affiliations:** 1 Department of General Surgery, Faculty of Medicine, Eskisehir Osmangazi University, Eskişehir, Turkey; 2 Department of Radiology, Eskisehir Osmangazi University, Eskişehir, Turkey

**Keywords:** Hepatic artery variations, Pancreaticoduodenectomy, Whipple procedure

## Abstract

Variations in the hepatic arterial system, observed in up to 22% of the population, significantly influence the outcomes of hepatobiliary and pancreatic procedures. This study describes an extremely rare variant involving an aberrant right posterior hepatic artery (aRPHA) originating from the gastroduodenal artery (GDA) alongside a replaced left hepatic artery (LHA). Awareness of such variants is critical during pancreaticoduodenectomy to prevent ischemic complications.

## Purpose

Variations in the hepatic arterial system, observed in up to 22% of the population, significantly influence the outcomes of hepatobiliary and pancreatic procedures [1, 2]. This study aims to describe an extremely rare variant involving an aberrant right posterior hepatic artery (aRPHA) originating from the gastroduodenal artery (GDA) alongside a replaced left hepatic artery (LHA).

## Methods

**Fig. 1:**
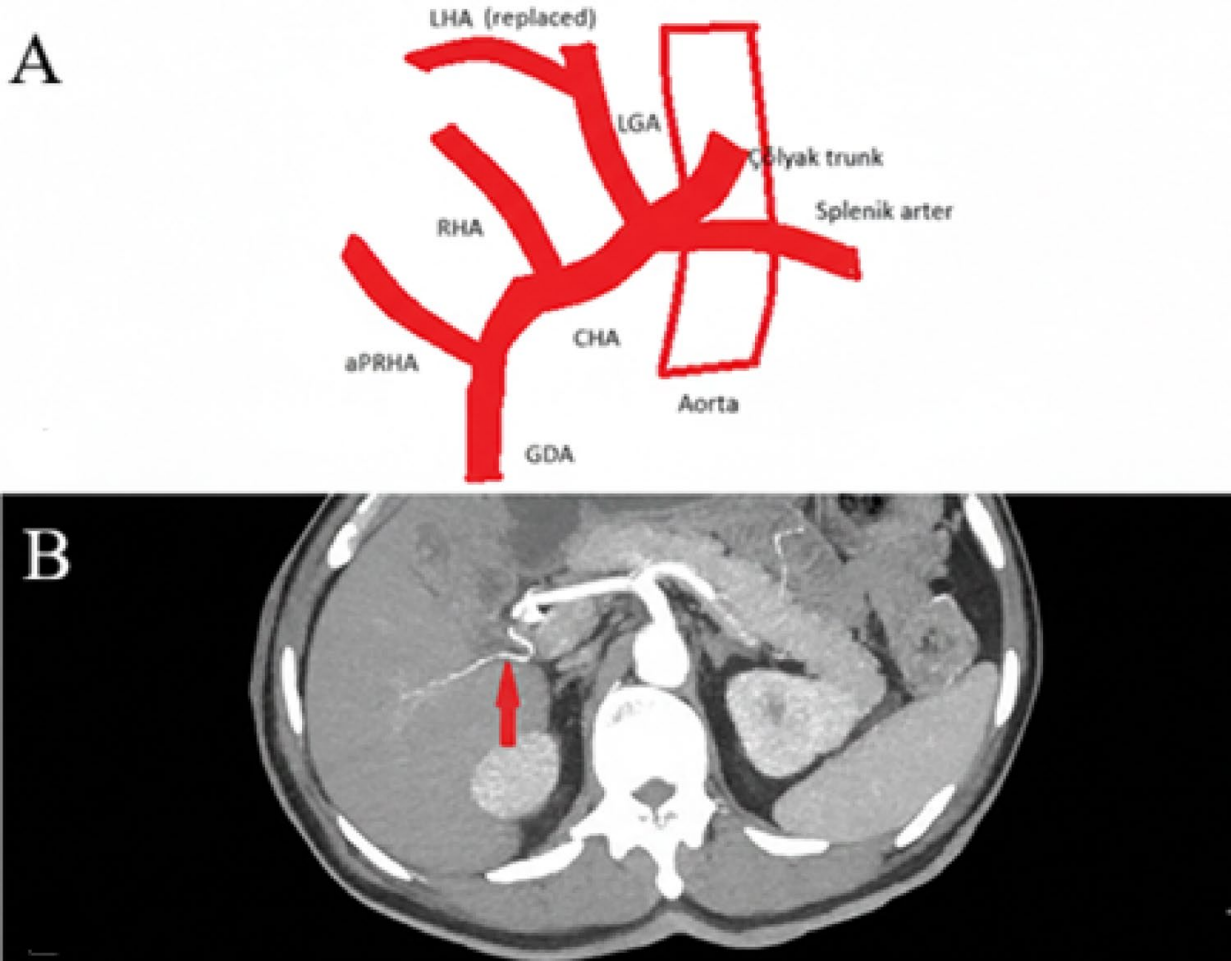
**A** Schematic illustration of the hepatobiliary arterial variations identified in this case, including the aRPHA from the gastroduodenal artery and a concomitant replaced left hepatic artery from the left gastric artery. **B** Axial CT angiography demonstrating the aberrant right posterior hepatic artery (aRPHA) branching from the gastroduodenal artery (arrow)

**Fig. 2:**
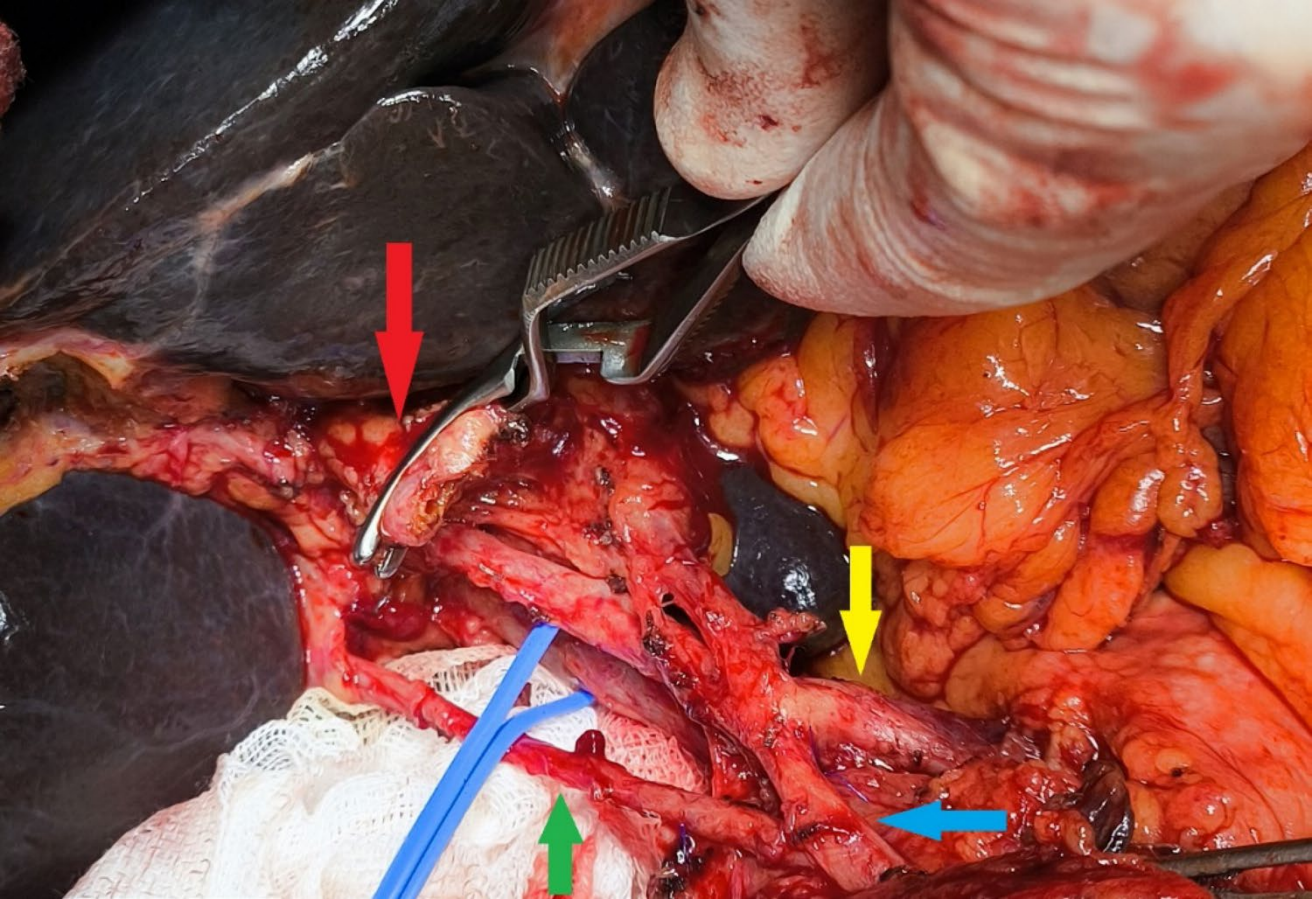
Intraoperative view during pancreaticoduodenectomy showing the preservation of the aRPHA after GDA identification

A 57-year-old male presenting with obstructive jaundice due to a pancreatic head mass was evaluated (Figs. 1, 2). Detailed preoperative vascular assessment was performed using axial CT angiography and 3D reconstructions. The findings were subsequently confirmed during a pancreaticoduodenectomy (Whipple procedure) [3].

## Results

Preoperative imaging revealed a dual vascular variant: an aRPHA arising directly from the GDA and a replaced LHA originating from the left gastric artery (LGA). During the surgical dissection, the aRPHA was identified and carefully preserved before the ligation of the GDA to prevent ischemia in the right posterior segments of the liver. The procedure was completed without vascular complications (Table 1). The patient’s recovery was uneventful, and no hepatic perfusion deficits were observed postoperatively.

**Table 1:** Comparison of the reported case with standard anatomical classifications

Feature	Standard anatomy	Michel / Hiatt Classification	Our case
**RPHA Origin**	Right Hepatic Artery	From SMA (Type III) or replaced	**From GDA (Unclassified)**
**LHA Origin**	Left Hepatic Artery	From LGA (Type II)	**From LGA (Replaced LHA)**
**Surgical Risk**	Low (GDA ligation safe)	Moderate	**High (GDA ligation risk)**

### Introduction and embryological basis

The hepatic arterial system develops from a complex arrangement of four primary vitelline arteries [2, 4]. During normal embryogenesis, the persistent communication between the celiac trunk and the superior mesenteric artery (SMA) through the longitudinal anastomosis usually regresses. The occurrence of an aRPHA from the GDA suggests a persistent primitive connection between the ventral splanchnic arteries and the hepatic bud during the longitudinal anastomosis formation [4, 5]. The coexistence of a replaced LHA indicates a developmental shift where the left hepatic bud’s blood supply remains linked to the left gastric artery instead of the common hepatic artery.

## Discussion

The anatomy of the hepatic arteries is notorious for its variability. While classic classifications by Michels and Hiatt provide a framework for most variations, they fail to account for the origin of major hepatic branches from the GDA [1, 2, 6]. The clinical significance of this variation is most pronounced during a pancreaticoduodenectomy (PD). In a standard PD, the GDA is routinely ligated. However, in our patient, the aRPHA—which supplied the posterior segments of the right lobe—branched directly from the GDA. Failure to recognize this unusual origin would have resulted in accidental ligation, leading to segmental hepatic ischemia or biliary fistula [7, 8]. The coexistence of both aRPHA and replaced LHA is exceptionally rare; for instance, Kobayashi et al. reported a frequency of only 0.2% for GDA-originating hepatic arteries in a series of 1200 cases [9]. Similar rare findings have emphasized the need for highly individualized preoperative mapping [10].

### Study limitations

This study describes an anatomical variation identified in a single clinical case. While MDCT angiography and intraoperative findings confirmed the variant, the lack of large-scale statistical data on this specific dual variation (aRPHA from GDA and replaced LHA) limits our ability to predict its prevalence across different ethnic populations. Nevertheless, the clinical relevance for pancreaticoduodenectomy remains paramount.

## Conclusion

Heightened awareness of rare variants, such as an aRPHA originating from the GDA, may serve as a critical factor in enhancing surgical safety and reducing the risk of unintended ischemic complications during complex procedures like the Whipple surgery. While our findings are limited to a single clinical case, they underscore the importance of meticulous preoperative imaging and intraoperative vigilance to prevent vascular injury in the presence of rare hepatic arterial variations [3, 4].

## Data Availability

No datasets were generated or analysed during the current study.
